# CYP1B1 G199T Polymorphism Affects Prognosis of NSCLC Patients with the Potential to Be an Indicator and Target for Precise Drug Intervention

**DOI:** 10.1155/2017/1529564

**Published:** 2017-03-09

**Authors:** Fengzhou Li, Shaofeng Zhang, Qi Zhang, Jinxiu Li, Shilei Zhao, Chundong Gu

**Affiliations:** ^1^Department of Thoracic Surgery, The First Affiliated Hospital of Dalian Medical University, Dalian, Liaoning 116011, China; ^2^Lung Cancer Diagnosis and Treatment Center, Dalian, Liaoning 116011, China; ^3^Department of Thoracic Surgery, Xingtai People's Hospital, Xingtai, Hebei 054031, China; ^4^China National Research Institute of Food & Fermentation Industries, Beijing 100015, China

## Abstract

CYP1B1 gene single nucleotide polymorphisms G119T, C432G, and A453G were tested among 164 NSCLC patients treated by Video-Assisted Thoracoscopic Surgery. After a follow-up period of 5 years, it was found that CYP1B1 G119T mutant genotypes were related to a higher risk of tumor recurrence and death after surgical resection. However, C432G and A453G genotypes had no influence on long-term prognosis of the study cohort. Thus, G199T alleles are supposed to be an auxiliary predictor for prognosis of NSCLC patients and a potential target for precise drug intervention, as well as a candidate for further anticancer drug research.

## 1. Introduction

Lung cancer is the most common malignancy and cause of cancer death in China, as was claimed in the latest epidemiological report published by the National Cancer Center in 2015 [1]. Non-small cell lung cancer (NSCLC) accounts for approximately 85% of all lung cancers, and surgical operation is the major treatment method [2]. Along with medicine unceasing progress, the diagnosis and treatment methods of NSCLC were improved dramatically. However, the 5-year survival rate of NSCLC patients remains as low as 15~16% [3, 4]. Drug adjuvant therapies such as chemotherapy, molecular targeting treatment, or herbal therapies [5] are often required in order to improve the overall survival of those patients with high recurrence risks. Accurate and efficient predictors for NSCLC postoperative prognosis as well as potential targets for precise drug interventions are still needed [6].

Cytochrome P450 (CYP450), a superfamily of enzymes, participates in the metabolism of many xenobiotic compounds and endogenous lipophilic substances [7]. To date, 57 CYP450 enzymes were found in human, most of which were involved in the biosynthesis of essential sterols, signaling molecules, and regulatory factors, while some potential functions remain unclear [8]. CYP1B1 (UniGene ID Hs.154654) as the only member of CYP1B gene family was first cloned from a human keratinocyte line in 1994 [9] and confirmed in regulating the metabolic activation [10]. Simultaneously, CYP1B1 protein was observed overexpressed in the livers of patients in many cancers (including lung cancer [7]), which was strongly implying that CYP1B1 may have relevance with cancers process.

The human CYP1B1 gene is located on chromosome 2p22-21 spanning approximately 12 kilobases (kb) of DNA and is composed of three exons and two introns [11]. To our knowledge, 6 CYP1B1 SNPs had been discovered and 4 of them could lead to amino acid substitution [12]. Further, CYP1B1 SNPs have been concerned with the occurrence of various types of cancer [13–19], including lung, breast, endometrium, prostate, bladder, liver, cervix, and colorectum. Among all of the CYP1B1 SNPs, the best studied were C432G (Leu 432 Val, rs1056836) and A453G (Asn453Ser, rs1800440) on exon 3 [20]. In the current research, we analyzed genotypes of G119T (Ala119Ser, rs1056827) codon on exon 2 and C432G (Leu 432 Val) and A453G (Asn453Ser) on exon 3 [13], to explore the relevance between CYP1B1 genetic SNPs and postoperative prognosis of NSCLC patients.

## 2. Material and Methods

### 2.1. Study Population

A total of 164 consecutive NSCLC patients treated by Video-Assisted Thoracoscopic Surgery (VATS) in the Department of Thoracic Surgery, the 1st Hospital of Dalian Medical University, Liaoning Province, China, during June 2011 to June 2012 were enrolled in the study. Baseline data such as gender, age, and smoking history were recorded by the initial questionnaire. All of those patients accepted conventional three-port VATS lobectomy by the same surgeon and had completed postoperative pathological reports showing the pathological types, pTNM stages, clinical stages, and the degrees of tumor differentiation. Prognostic data such as recurrence and survival status was recorded by regular postoperative rechecks and calling back interview, and the end point of the follow-up was 61 months after the first operation (48 months after the last operation). The TNM stages and clinical stages were defined according to the Eighth Edition of the IASLC TNM Classification for Lung Cancer [21–23]. Those patients who reportedly had previous cancer, other metastasized tumors, and preoperative radiotherapy or chemotherapy were excluded. This research was also approved by the local ethics committee, and the informed consent form according to the Declaration of Helsinki was obtained by each subject.

### 2.2. DNA Isolation and Genotyping Assays

**Figure 1:**
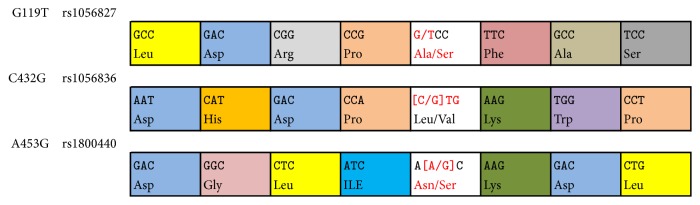
Mode chart of CYP1B1 G199T, C432G, and A453G SNPs.

Mode chart [24] of CYP1B1 G199T, C432G, and A453G SNPs was shown in Figure 1. Peripheral venous blood samples (2 mL) from each patient were collected before surgery in EDTA tubes and were quickly put into the liquid nitrogen tank. Then the samples were transferred into the lab and were stored in −80°C for DNA isolation. Genomic DNA was extracted from the blood samples by using the TaKaRa Blood Genome DNA Extraction Kit, and extraction process was referred to the recommended protocol. SNP genotyping was performed using polymerase chain reaction-sequence specific primer technique (PCR-SSP). Primers and product length were designed according to previous literature (Table 1) [12]. The PCRs were performed on 94°C for 3 min to degeneration, followed by 30 cycles of 94°C for 30 s, 56°C for 30 s, and 72°C for 30 s, and a final extension at 72°C for 5 min. The extended chains were cut by BsrI and MwoIs restriction enzyme. Genotype of each subject was finally detected by FluorChem FC2 UV transmission imaging system. Each SNP found was tested for deviation from Hardy–Weinberg equilibrium using SNPstats and Haploview [25, 26].

### 2.3. Statistical Analysis

Statistical data was analyzed using IBM Statistical Product and Service Solutions 22.0 (SPSS 22.0). Differences of clinical characteristics across genotypes were evaluated using Chi-square test and Fisher exact probability test. In those variables existing intergroup differences, Fragmentation Independence was performed by Bonferroni method to compare column proportions. To compromise the heterogeneity of the follow-up periods, survival curves were plotted with Kaplan-Meier survival analysis [27], and the differences between those curves were analyzed by the Log-Rank Test [28]. The Cox's proportional hazards model was applied to analyze the significance and independence of the influence of all the studied clinical and pathological factors on both tumor recurrence and cancer death risks during the follow-up period [29]. As dummy variables were established to analyze polytomous variables, the method of variables entering the Cox's equation was limited to the ‘Enter' method, which means all the variables entered the equation at the same time. The difference was considered to be significant at *p* < 0.05.

## 3. Results

### 3.1. Differences of Pathological and Clinical Factors across Genotypes

Differences of clinical characteristics across genotypes were shown in Table 2. 100 (61%) male and 64 (39%) female NSCLC patients were included in the study. The mean age was 64.4 ± 11.5 (median = 66, ranging from 26 to 83). Genotype frequencies were shown in Table 2. In the total of 164 subjects, we detected three genotypes for G119T codon, including the wild type (G/G) in 97 patients and two mutant types (G/T and T/T) in 60 and 7 patients, respectively. For C432G codon, we also detected three genotypes, including the wild type (C/C) in 124 patients and two mutant types (C/G and G/G) in 35 and 5 patients, respectively. However, we could only detect the A/A genotype for A453G codon in most subjects, while A/G, a rare variant of CYP1B1 gene A453G codon, was found in 2 patients. For A453G codon, G/G variant was not found in the study population. These findings implied that there might not exist statistically significant SNP of CYP1B1 gene A453G codon in the Chinese population. When assessing each SNP, patients were divided into two groups by wild or mutant genotypes. Chi-square test and Fisher exact probability test were used to evaluate intergroup difference. Among all the included clinical and pathological factors, only the degree of tumor differentiation (*χ*^2^ = 11.176, *p* = 0.004) and pathological T stage (*χ*^2^ = 9.448, *p* = 0.024) caused significant difference across G199T genotypes. None of the variables showed intergroup difference across C432G genotypes.

### 3.2. Survival Analysis

**Figure 2:**
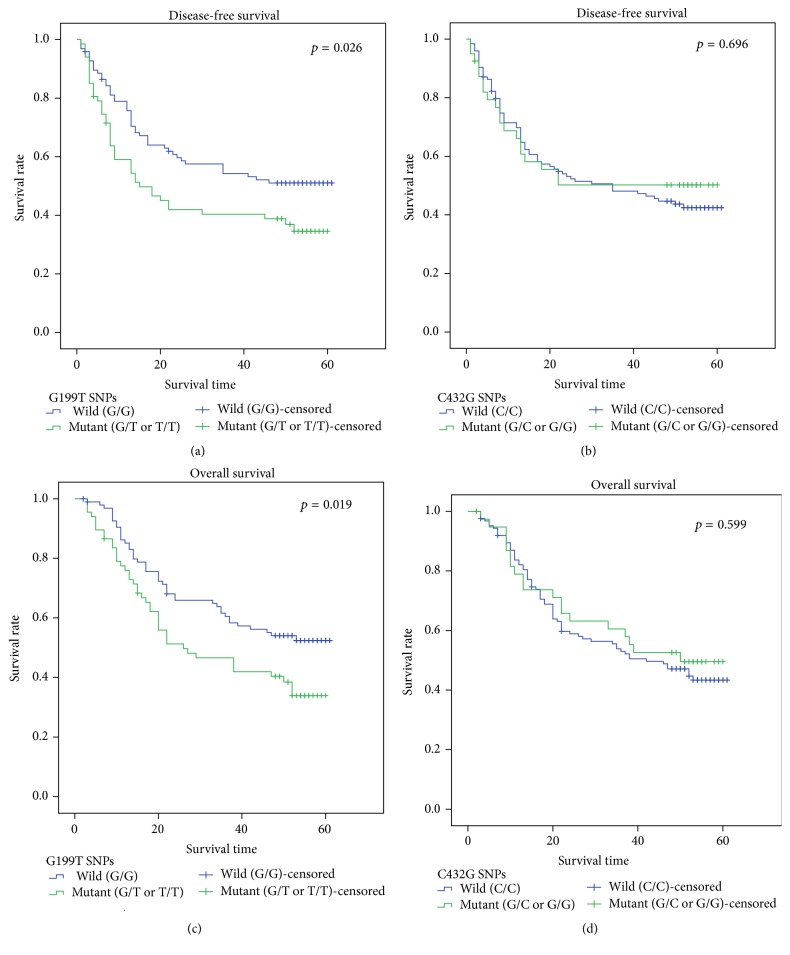
Kaplan-Meier survival analysis for disease-free and overall survival time.

Within the complete follow-up period, 88 patients (53.7%) had recurrences (mean = 9 months, ranging from 0 to 53 months). 86 patients (52.4%) in those who had recurrence were killed. Three patients were killed by other reasons. Kaplan-Meier survival analysis was used to describe the survival curves of both overall and disease-free survival status (Figure 2). We found that patients with G119T wild type (G/G) had benefits in both disease-free (*p* = 0.26) and overall (*p* = 0.19) survival time compared with those carrying mutant types (G/T or T/T). However, there was no significant difference in overall nor disease-free survival time between patients with C432G wild type (C/C) and mutant types (C/G or G/G, *p* < 0.05). Besides, a Cox's proportional hazard regression model was performed. Univariate analysis (Table 3) showed that, along with pathological types, tumor differentiation, pTNM stages, and clinical stages, G199T polymorphism was an influence factor of both recurrence (HR = 1.592, 95% CI 1.047–2.420, *p* = 0.03) and cancer death (HR = 1.640, 95% CI 1.074–2.505, *p* = 0.022) risks, while C432G genotype was not. Further, in multivariate analysis (Table 4), we found that pathological types, clinical staging, and pTMN staging were still significant influence factors of NSCLC long-term risks, but neither G119T nor C432G genotype could be an independent predictor for prognosis.

## 4. Discussion

During the last 30 years, the diagnosis and treatment of lung cancer had undergone great improvement; however the prognosis remained optimistic [30]. Even for stage I NSCLC patients, approximately 30% of patients would have recurrences of the tumor and die despite complete surgical resection [31]. The prognosis of lung cancer is associated with multiple factors, such as surgery technology, perioperative management, degree of tumor differentiation, pTNM stage, clinical stage, and postoperative therapies [32]. Besides, there have been numerous studies on genetic SNPs as the predictors of prognosis in patients with NSCLC after surgical resection. For example, in 2015, Lee [33] and colleagues identified 8 human SNPs significantly associated with NSCLC prognosis, including CD3EAP rs967591, TNFRSF10B rs1047266, AKT1 rs3803300, C3 rs2287845, HOMER2 rs1256428, GNB2L1 rs3756585, ADAMTSL3 rs11259927, and CD3D rs3181259. Chen [34] and colleagues reported VEGF rs3025039 polymorphism could influence the response to chemotherapy and overall survival of NSCLC patients. Most SNPs, which were found to affect NSCLC prognosis, belonged to genes encoding important proteins in the cancer process.

The occurring and development of cancer are associated with abnormity of multiple cancer-related genes, among which CYP1B1 acts as an important phase I metabolism enzyme participating in regulating the metabolic activation [35]. Over the last two decades, a number of case-control studies were conducted to investigate the association between CYP1B1 gene polymorphism and cancer risk in humans [13–19]. Researchers considered that it was the SNPs which led to amino acids substitution resulting in conformational variation of the CYP1B1 protein and thus causing downregulation of activity or change in function of the enzyme. This change might weaken the capability on procarcinogen metabolism of body and therefore increased susceptibility to cancer [16, 17]. Besides, numerous researches believed that the constituent ratio of CYP1B1 polymorphisms possesses regional and ethnic differences [10, 36–39]. In the current study, the constituent ratio of each enrolled SNP was in accordance with previous reports about other Chinese populations (there were also some Chinese written articles in domestic journals giving similar reports) [10, 37, 40]. For CYP1B1 A453G codon, we did not find statistically significant SNPs among the study groups. This finding was in accordance with most researches enrolling A543G codon among Chinese, suggesting that there did not exist A453G SNPs in Chinese Han population.

CYP family was well known to be associated with drug metabolism [41]. Lots of CYP superfamilies such as CYP1A, CYP2C, CYP2E, CYP2D, and CYP3A [42–44] appeared to have mutual effect with numerous drugs. However, the role of CYP1B superfamily in drug metabolism was rarely studied, especially for anticancer drugs. Before us, there was no report on the influence of CYP1B1 gene polymorphisms on lung cancer prognosis. As some reports said that downregulation of P450 enzymes expression is associated with tumor progression [45], we assume that the changes of enzyme activity or function caused by CYP1B1 gene SNPs could either have immediate influence on tumor process or cause boost or block in drug metabolism which could indirectly affect tumor process and therefore affect long-term survival conditions. Among all of the 164 NSCLC cases, the study showed those patients carrying G199T mutant (G/T or T/T) genotypes suffered from higher expectation of recurrence and shorter survival time. Although the result of Cox's model multivariate analysis implied that the polymorphism of G199T could not independently affect NSCLC long-term prognosis, those patients with G199T mutant genotypes (G/T or G/G) tend to have later T stages and poorer tumor differentiation, which were confirmed as independent risk factors of recurrence and cancer death. This finding implied that although G199T SNP cannot act as an independent predictor for NSCLC postoperative prognosis, it could be an auxiliary predictor. It is implied that patients carrying G199T mutant genotypes might had worse clinical and pathological conditions, as well as lower expectation of disease-free and overall survival time, who perhaps need additional adjuvant therapy. However, we still know little about the exact mechanism of CYP1B1 gene SNPs interacting with tumor development. We assumed that it was also the shift in enzyme activity or function caused by base-pair substitution that affected tumor progress.

## 5. Conclusion

This study showed CYP1B1 gene G199T SNP could be useful to identify patients with a higher risk of tumor recurrence and death after surgical resection of NSCLC and thereby help to select patients for preoperative or postoperative drug adjuvant therapies. Besides CYP1B1 G199T alleles could also be a potential target for precise drug intervention, as well as a candidate for further research in the field of anticancer drugs. Studies are required to confirm the validity of this SNP in other ethnic populations, and the mechanism of CYP1B1 genetic polymorphism affecting cancer progress should be explored in vitro and in vivo.

## Figures and Tables

**Table 1:** Primers design and product length for PCR-SSP.

		Product length

	C432G	
Upstream	5′-ATGCGCTTCTCCAGCTTTGT-3′	
Downstream 1	5′-TCCGGGTTAGGCCACTTCAG-3′	175 bp
Downstream 2	5′-TCCGGGTTAGGCCACTTCAC-3′	

	G119T	
Upstream	5′-ATGCGCTTCTCCAGCTTTGT-3′	
Downstream 1	5′-TCTGCTGGTCAGGTC-CTTGT-3′	347 bp
Downstream 2	5′-TCTGCTGGTCAGGTCCTTGC-3′	

	A453G	
Upstream	5′-ATGCGCTTCTCCAGCTTTGT-3′	
Downstream 1	5′-TCTGCAGGTCCTGGTCTTGC-3′	328 bp
Downstream 2	5′-TCTGCAGGTCTGGTCCTTGT-3′	

**Table 2:** Differences of clinical characteristics across genotypes.

Clinical factors	Total (%)	G119T				C432G			
		G/G	G/T&T/T	*χ* ^2^	*p*	C/C	C/C&G/G	*χ* ^2^	*p*
Age				0.461	0.302_f_			1.174	0.184_f_
≥66	86 (52)	53	33			68	18		
Gender				1.050	0.297_f_			0.269	0.368_f_
Male	100 (61)	56	44			77	23		
Tobacco use				0.041	0.483_f_			0.016	0.526_f_
Ever	67 (41)	39	28			51	16		
Pathological type				1.783	0.619			6.476	0.091
AD	78 (48)	44	34			52	26		
SQ	56 (34)	37	19			47	9		
LA	19 (12)	10	9			16	3		
Others	11 (7)	6	5			9	2		
Differentiation				**11.176**	**0.004**			0.970	0.616
Well	50 (30)	20_a_	30_a_			39	11		
Moderate	89 (57)	59_b_	30_b_			68	21		
Poor	25 (15)	18_b_	7_b_			17	8		
pTNM									
T				**9.448**	**0.024**			5.657	0.130
T1	36 (22)	27_a_	9_a_			30	6		
T2	60 (37)	36_a,b_	24_a,b_			49	11		
T3	45 (27)	26_a,b_	19_a,b_			30	15		
T4	23 (14)	8_b_	15_b_			15	8		
N				1.287	0.732			1.320	0.724
N0	81 (49)	45	29			55	19		
N1	31 (19)	8	15			24	9		
N2	49 (30)	33	21			42	12		
N3	3 (2)	1	2			3	0		
M				0.076	0.477_f_			0.102	0.490_f_
M0	141 (86)	84	57			106	35		
M1	23 (14)	13	10			18	5		
Clinical stages				5.206	0.267			1.232	0.873
I	51 (31)	32	19			36	15		
II	16 (10)	9	7			13	3		
IIIa	59 (36)	38	21			45	14		
IIIb	15 (9)	5	10			12	3		
IV	23 (14)	13	10			18	5		

^a, b^Each subscript letter denotes a subset of the variate categories whose column proportions do not differ significantly from each other at the 0.05 level, as were calculated by Bonferroni method.

^f^The  *p* value was adjusted by Fisher's exact probability.

AD, adenocarcinoma; SQ, squamous carcinoma; LA, large cell carcinoma; T, tumor staging; N, lymph node staging; M, metastasis staging.

**Table 3:** Cox's proportional hazard regression model for univariate analysis.

Variables	Disease-free survival				Overall survival			
	*p*	Exp (B)/HR	95% CI		*p*	Exp (B)/HR	95% CI	
			Lower	Upper			Lower	Upper
Gender (male)	0.119	1.425	0.913	2.224	0.165	1.377	0.876	2.162
Age (≥66)	0.710	0.924	0.608	1.404	0.648	0.906	0.593	1.383
Smoking (never)	0.807	1.054	0.690	1.610	0.777	1.064	0.694	1.630
Pathological type								
AD	**0.017 **	1.000			**0.019 **	1.000		
SQ	0.164	0.707	0.433	1.153	0.266	0.754	0.459	1.240
LA	**0.040 **	1.877	1.028	3.428	**0.024 **	2.005	1.096	3.669
Others	0.209	0.519	0.187	1.444	0.269	0.561	0.202	1.564
Differentiation								
Poor	**0.012 **	1.000			**0.004 **	1.000		
Moderate	0.119	0.701	0.449	1.096	0.124	0.704	0.451	1.101
Well	**0.004 **	0.298	0.131	0.675	**0.001 **	0.212	0.082	0.543
T stage								
T1	**0.001 **	1.000			**0.001 **	1.000		
T2	**0.002 **	3.265	1.571	6.788	**0.000 **	0.187	0.083	0.422
T3	**0.008 **	2.773	1.298	5.924	0.076	0.590	0.329	1.057
T4	**0.000 **	5.196	2.305	11.713	0.082	0.580	0.315	1.071
N stage								
N0	**0.000 **	1.000			**0.000 **	1.000		
N1	**0.017 **	2.073	1.140	3.768	**0.004 **	2.447	1.331	4.499
N2	**0.000 **	3.197	1.938	5.276	**0.000 **	3.589	2.146	6.002
N3	**0.002 **	7.186	2.117	24.396	**0.001 **	7.839	2.318	26.516
M stage (M1)	**0.000 **	2.538	1.526	4.221	**0.001 **	2.484	1.478	4.174
Clinical stage								
I	**0.000 **	1.000			**0.000 **	1.000		
II	**0.003 **	3.513	1.538	8.025	**0.001 **	3.923	1.693	9.092
IIIa	**0.001 **	2.844	1.500	5.394	**0.001 **	3.194	1.653	6.173
IIIb	**0.000 **	5.266	2.348	11.810	**0.000 **	5.608	2.466	12.751
V	**0.000 **	5.896	2.899	11.989	**0.000 **	6.246	3.002	12.997
G199T (mutant)	**0.030 **	1.592	1.047	2.420	**0.022 **	1.640	1.074	2.505
C432G (mutant)	0.700	0.905	0.545	1.504	0.603	0.874	0.525	1.454

**Table 4:** Cox's proportional hazard regression model for multivariate analysis.

Variables	Disease-free survival				Overall survival			
	*p*	Exp (B)/HR	95% CI		*p*	Exp (B)/HR	95% CI	
			Lower	Upper			Lower	Upper
Gender (male)	0.088	1.679	0.926	3.045	0.265	1.400	0.775	2.529
Age (≥66)	0.180	1.412	0.852	2.339	0.102	1.539	0.918	2.580
Smoking (never)	0.088	0.623	0.362	1.074	0.082	0.608	0.347	1.064
Pathological type								
AD	**0.014 **	1.000			**0.001 **	1.000		
SQ	0.137	0.624	0.335	1.162	0.230	0.674	0.354	1.284
LA	**0.050 **	2.018	0.999	4.078	**0.005 **	2.834	1.371	5.859
Others	0.642	0.775	0.264	2.273	0.871	1.096	0.363	3.306
Differentiation								
Poor	0.139	1.000			0.028	1.000		
Moderate	0.256	0.753	0.462	1.228	0.155	0.700	0.428	1.144
Well	0.055	0.412	0.167	1.018	**0.009 **	0.255	0.091	0.712
T stage								
T1	**0.002 **	1.000			**0.001 **	1.000		
T2	**0.000 **	4.051	1.858	8.835	**0.000 **	4.320	1.978	9.435
T3	**0.040 **	2.422	1.040	5.640	**0.007 **	3.171	1.379	7.290
T4	**0.003 **	5.524	1.783	17.109	**0.001 **	7.859	2.425	25.467
N stage								
N0	0.161	1.000			**0.031 **	1.000		
N1	0.572	0.763	0.299	1.948	0.932	1.043	0.398	2.734
N2	0.170	1.659	0.805	3.418	**0.014 **	2.613	1.212	5.630
N3	0.313	2.236	0.469	10.667	0.215	2.681	0.565	12.719
M stage (M1)	**0.001 **	5.499	2.079	14.543	**0.002 **	4.708	1.746	12.689
Clinical stage_a_								
I	**0.049 **	1.000			0.115	1.000		
II	**0.005 **	6.274	1.725	22.816	**0.018 **	4.878	1.316	18.076
IIIa	0.089	2.312	0.881	6.064	0.302	1.701	0.621	4.658
IIIb	0.219	2.413	0.593	9.824	0.522	1.595	0.383	6.647
G199T (mutant)	0.491	1.176	0.742	1.863	0.478	1.185	0.741	1.896
C432G (mutant)	0.536	1.200	0.674	2.138	0.665	1.137	0.637	2.30

^a^Constant or linearly dependent covariates clinical stage V = M stage 1.
